# Head to head searching: the Search&Destroy card game in the health sciences classroom

**DOI:** 10.5195/jmla.2026.2299

**Published:** 2026-10-01

**Authors:** Lena Bohman, Regina Vitiello, Christiana M. Keinath, Gary R. Maixner, Mari Kermit-Canfield

**Affiliations:** 1 lena_bohman@rush.edu, Assistant Professor, Senior Data Services and Research Impact Librarian, Zucker School of Medicine at Hofstra/Northwell, Hempstead, NY; 2 rvitiello1@northwell.edu, Librarian, North Shore University Hospital, Northwell Health, Manhasset, NY; 3 keinath@etsu.edu, Health Sciences Librarian, Assistant Professor, Charles C. Sherrod Library, East Tennessee State University, Johnson City, TN; 4 gmaixner@iu.edu, User Experience Librarian and Liaison to Communication Studies, University Library, Indiana University Indianapolis, Indianapolis, IN; 5 kermitt@ferris.edu, Creative Learning Librarian and Coordinator of Research Services, Professor, FLITE Library, Ferris State University, Big Rapids, MI

**Keywords:** Game-based learning, games, card games, game design, gamification, game development

## Abstract

**Background:**

Games are a promising way to make health sciences education more engaging and improve synthesis for learners. There have been some successful implementations of game-based learning for health sciences library instruction [1], but there is room for development of games tailored specifically for teaching database searching skills to health sciences students.

**Case Presentation:**

The authors developed a biomedical edition of the existing library instruction card game Search&Destroy. The original Search&Destroy was developed as an alternative way to teach a one-shot instructional session on search skills using a discovery layer in an academic library for undergraduates. The biomedical edition modified the game to work with PubMed and incorporate health science-specific searching skills. Keyword cards were updated with biomedical terms and new cards were added with filters and actions related to health sciences literature searching (e.g., last five years, systematic reviews, randomized controlled trials filters). The new version was play tested internally by the authors and then piloted in classes with nurse residents. The game is now available through the print-on-demand service The Game Crafter and teaching materials are available online.

**Conclusions:**

Feedback from our pilot test group of nurse residents was overwhelmingly positive. Informal conversations with nurse educators responsible for the program were also positive, and the game has been incorporated in the curriculum for the future. The Biomedical Edition joins a growing family of Search&Destroy games designed for a variety of library instruction needs.

## BACKGROUND

### Problem Statement

Health sciences education is still a relatively traditional field, with slides and large group lectures being the predominant format [2, 3, 4], despite growing attempts at reform [5]– for example, incorporating more case-based learning and flipped classroom formats [6]. This has also permeated health sciences library instruction on literature searching [7, 8, 9].

As librarians, many times our classroom experience is limited to one-shot instruction. The one-shot instruction session is limiting, often forcing the librarian to choose between searching skills instruction or content evaluation in a short amount of time and commonly in an exhausting (and boring) lecture setting. Moreover librarians typically can’t spend the same amount of time cultivating the trust and respect of students that their instructors do. Game based learning presents an alternative and an opportunity to use games for the same benefits they provide outside of an educational setting [10].

The authors wanted to take advantage of the increasing openness of health sciences education to new teaching methods and try a game-based learning intervention to increase student engagement in library literature searching instruction. One way to innovate on health sciences library instruction is to borrow ideas from academic librarians, who have historically had more leeway to try new teaching modes [11].

### Literature Review

#### Game-Based Learning as a Solution for Student Engagement

The power of games to hold attention and inform has been discussed at length in game design literature and game studies Gamification in particular is widespread, whether it be loyalty points at a grocery store or hotel [12], collecting digital badges [13], or with spinning wheels and games of chance in online marketplaces [14]. This benefits educators to more easily implement gamification, as students are familiar with game concepts [15,16]. This prior experience encourages quicker adoption and reduces learner frustration. Because gamification and game-based learning are incorporated into many aspects of modern life, students are often familiar with game mechanics regardless of whether they know these terms or are interested in board games or video games.

Game design is primarily studied through the lens of positive psychology. The core concept of positive psychology is that focus and engagement comes from a match between a person’s skill and the challenge of an activity. If these two are not in harmony, the person will become bored, anxious, or frustrated [17]. Through this model, we can view games as tools to modify the difficulty of a task, making them perfect for changing students’ perceptions of subject matter [18].

It is through this positive psychology framework that game-based learning is used, seeking to create games that impart learning outcomes through the act of play, rather than using game elements only to increase motivation and task completion [19]. By directly connecting learning and play in this way, instructors are able to create bespoke, meaningful experiences for students.

#### Game-Based Learning in Health Sciences Education

A growing body of literature examines the use of games in health sciences education. Recent systematic reviews suggest that games can improve learning outcomes, such as knowledge acquisition, and learner engagement [10, 20, 21, 22]. Analog games, such as board and card games, have a history of use with healthcare trainees [23]. Digital serious games (games where-in the designer’s goal is beyond creating an entertaining experience), often incorporating new technologies like Artificial Intelligence (AI), Virtual Reality (VR), and Augmented Reality (AR), can show positive effects on learner knowledge, skills, and confidence [24, 25, 26].

However, the efficacy of games compared to other educational interventions is not consistent. A recent meta-analysis showed an overall inconclusive effect on learner skills, attitudes, and behavior [27]. Limitations such as small sample sizes, lack of rigorous control groups, and inconsistent reporting of theoretical frameworks mean that further research is necessary to improve game design, implementation, and evaluation [1, 28, 29, 30]. While the use of games in health sciences education continues to expand, there is still a need for more examples of games being incorporated into existing curricula and healthcare environments [31]. There have been two studies implementing game-based learning for literature searching and information literacy that demonstrated learner skill improvement [32, 33]. This successful use of games to improve skills for instructional topics relevant to libraries shows that there are potential benefits from incorporating games-based learning into library instruction.

Health sciences libraries are well positioned to champion game-based learning within their institutions. Games have the potential to support specialized clinical and research skills training [34]. Teaching strategies like escape rooms are one-way games can be adapted across different learning environments for learner needs [35]. With some training, librarians can partner with health sciences educators to develop, implement, and assess these learning experiences. It is possible for Library and Information Science programs to provide these instructional design skills [36]. An interactive special workshop, at the 2018 Medical Library Association annual meeting, successfully trained librarians to create and implement low-tech games for health sciences library instruction [37]. Through collaboration and with guidance, health sciences libraries can integrate games into existing curricula and spaces to improve the learning experience of healthcare professionals.

Card games provide a readily accessible, low-tech, and familiar format for educational games. A 2024 scoping review found that card games were the second most common format used in analog game-based learning in medical education [23]. A 2007 review identified various card games used in medical education, covering diverse topics [38]. Recent examples of medical education card games include the “Pit Crew Card Game” for improving EMS performance [39], “CARBGAME” for teaching clinical enzymology [40], and a game for teaching anatomy of the liver [41]. These examples demonstrate how card games can be used for teaching both medical concepts and practical skills. Card games focused on specific topics, like white blood cells [42] or viral hepatitis [43], show their use for specialized learning and knowledge reinforcement.

## CASE PRESENTATION

### Game Play of Search&Destroy

Search&Destroy is played in groups of 4–6 players, with each person accessing a library database. Students use cards to build search strings with keywords and place limiters (or facets) on one another, performing a search in a library database every turn. This search takes place in the actual discovery layer or database, thereby making the game immediately relevant to the students’ research. Players continue until they get zero results, causing them to lose, one-by-one. Typically, players last 3–4 rounds of play, so every student is getting to practice with the database several times and will watch their classmates do the same.

To set up the game, you need 4–6 players, with each player able to reach the cards. They also need space in front of them to place the cards that make up their searches. Each student will also need to access the library database. Ideally, the students can gather around a single computer and web browser, with each student having their own browser tab for their searches, but students can also play on their own laptops, phones, or tablets if needed.

There are three types of cards in the game:

1. Keyword Cards (68): These are cards with words or names that are combined to create the search string that students will use to search the research database
2. Play Cards (51): these are further broken down into two types of cards
  - Modification Cards (abbreviated to MOD Cards in game): These are limiters or facets that students will need to apply to their search strings
  - Action Cards: These cards are one time use cards that impact play directly. Some examples are that students may skip their next turn or they must draw entirely new keyword cards
3. Rules reference cards (6): These cards have no impact on gameplay, but explain the rules of the game in a truncated way so that the full rulebook does not need to be consulted during play

At the start of the game each player is given a rules reference card. The Keyword Cards are shuffled and placed face down to form the Keyword Deck, and the Play Cards are likewise shuffled to make their own deck. Then each student should draw four Play Cards to form their hand, but these should be kept secret.

After this set-up, the librarian teaches the students how to play with a live demonstration or with a presentation. This saves time and avoids confusion, instead of asking students to read the rules on their own.

Gameplay follows 5 steps (listed in Figure 1 below), which students must take in order. First the student will draw the top two cards from the Keyword Deck and choose one by placing it face-up in front of them, placing it on the end of all the other Keyword Cards they already have (if any). The other is discarded by placing face-up in the discard pile. Students are most successful if they choose their keywords strategically based on what they already have, to maximize the number of results the database returns.

**Figure 1:**
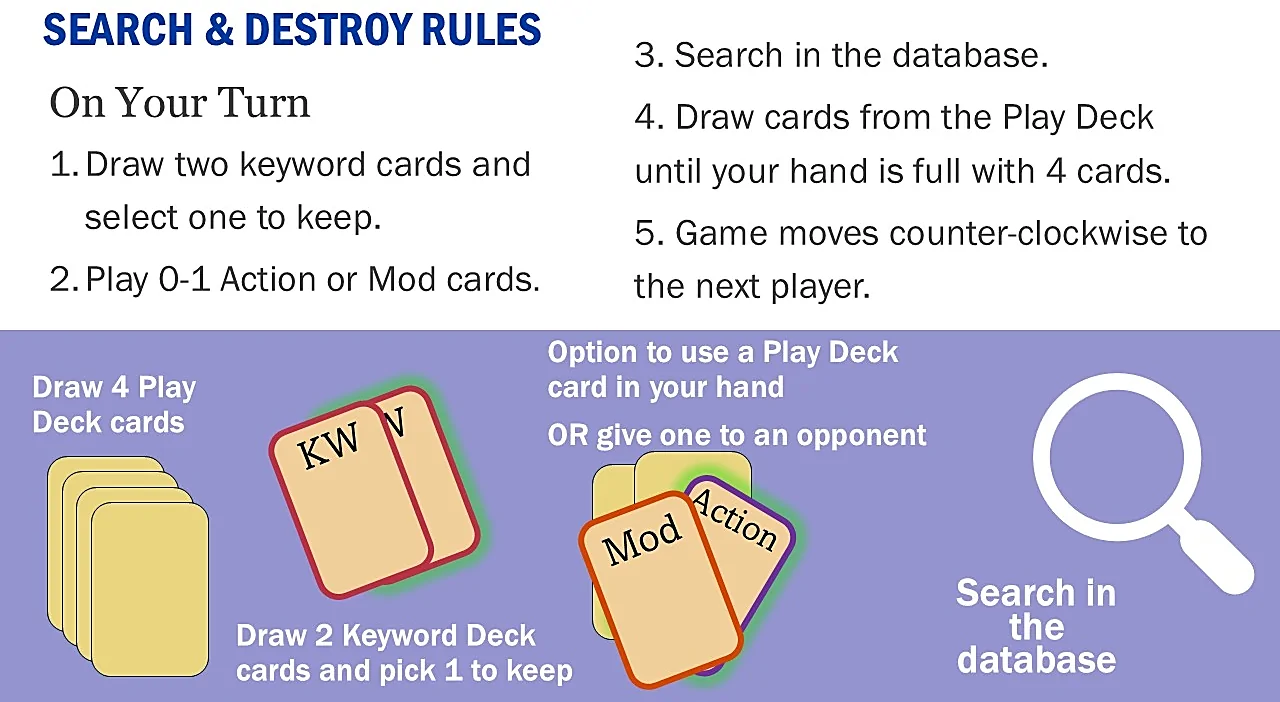
Search&Destroy rules summary.

The student then has the opportunity to play an Action or MOD Card. Action Cards are played once, do the effect listed on the card and then are discarded. MOD Cards are added to any student’s Keywords and must be included on their next search. These cards are what give students the most control of the game and have the most impact on their opponents. Some cards will make the game last longer (skipping turns or searches) or shorter (adding more keywords to a player's search, making a player filter their results to only French language). This step is skipped on each student’s first turn.

During step 3 the student will then take all the keywords in front of them and search them as a single string in the library database. This might look like, “rats AND naps AND Darwin,” or “asian AND carp AND star AND crown.” This is also where they should add the facets from MOD cards, such as “Items must be published before 1965” or “Items must be in French.” There is no limit to the number of keywords or facets that a student has to search. If the database returns zero results, the student’s turn ends and they are out of the game.

After searching, the player draws back up to four Play Cards and then passes the turn to the next player.

While rehearsing with a database is helpful, the real power of Search&Destroy is that to play effectively, a student must pay attention to their searches and the searches of their opponents. They are able to do this by playing Action or MOD cards, which change an opponent's search (e.g. “Results must be a Book”) or add more keywords to a student's search string. The incentive of winning makes all searches more immediately interesting to the players, even when it's not their turn.

Ultimately this makes Search&Destroy a fun and meaningful way to teach discovery level searching. It acts as an upgrade over other exercises that might be done class-wide, such as working through prepared example searches or asking students for topic ideas (favorite band, research topic, etc.).

### Development of Original Search&Destroy

The original edition of Search&Destroy was developed by Kermit-Canfield and Maixner as an alternative to teaching basic discovery layer search skills in one-shot classes at the collegiate level at Ferris State University. Ferris State is a public university in Big Rapids, MI, with 12,000 students. One-shot library classes can be challenging to teach, as students may be completely new to the academic world and library searching in general.

**Figure 2:**
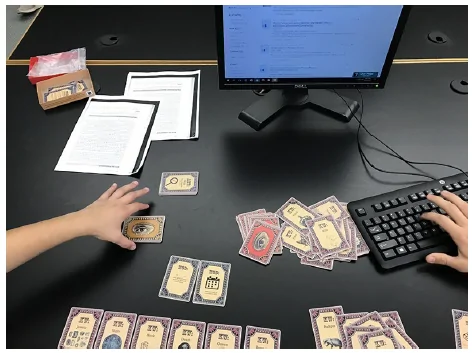
Playing the original Search&Destroy

Kermit-Canfield and Maixner’s goal was to fundamentally change the way they were teaching and interacting with students—to entirely replace classroom lectures with game-based learning. Learning outcomes for the game would include:

- Students will practice database use
- Students will employ limiters and facets in library databases
- Students will use Boolean operators to expand their searches
- Students will construct Search Strings for Database searching
- Students will identify and select library materials
- Students will recognize peer review as a concept

Game design is an iterative process - the designer must build a functional prototype, test it, take that feedback, and improve upon it. It is widely understood that a game will be refined by repeating this process as many times as possible [44]. In the case of Search&Destroy development started with a deck of keyword cards. From there, the designers sketched out the basics of the game and then competed against one another and then played with student workers to ensure that the game design was on the right track. After these initial tests more specific decisions about the game were made, such as the order of play, exactly what keywords would be included and how many cards a player interacts with at a time were made. A good example of this is in the opening of a turn, where a player draws two keyword cards and selects one to keep. In the very first tests of the game, players simply drew the top card, meaning there was no decision making at this step and keyword strings were completely random. The change to selecting a card was implemented as players felt that they had too little influence on their success in the game.

This design process is more challenging while maintaining the connection to learning outcomes - every action that a player can take should reinforce learning outcomes as much as possible, while still allowing space for fun and discovery by players. To this end, the designers made sure to include cards in the game that, while not directly reinforcing the learning outcomes, lead to fun moments. An example is the card, “Skip your Search,” an Action card that a student can play on themselves. This card allows the player to completely bypass the step in the game where they could be eliminated. Even though this card skips over the core learning outcome of the game (searching in the database), the designers found that adding options for players to disrupt the game was important to add more elements of competition and fun. Dialing the right ratio of “fun” elements to “learning” elements is something that can only properly be explored through playtesting, and updated editions to the game have adjusted these ratios after playing the game in class more often.

### Development of Biomedical Game Version

The idea for creating a biomedical edition of Search&Destroy came through conversation between the three health sciences librarians on the team (CK, LB and RV). East Tennessee State University was already using the original edition of Search&Destroy in its library instruction, and the librarians were inspired to try it out on PubMed to see its potential for health sciences library instruction. East Tennessee State University is an R2 state school with both nursing and medical schools in Johnston City, Tennessee. Northwell is a large health system made up of 27 hospitals in the New York City metro area that employs 80,000+ people. After testing, the basic mechanics of the game worked, but it needed modification to work well in a medical or health sciences setting.

Since the project seemed doable, the three health sciences librarians approached the original creators of Search&Destroy to gauge interest. Fortunately Kermit-Canfield was working on sabbatical to develop a Spanish language of the game and thought it was possible to work on a Biomedical Edition concurrently. It seemed that a collaboration was possible, with the health sciences librarians contributing knowledge of medical education and databases, to create a modified version. Librarians from other institutes have requested specific versions of the game (e.g. medical, science, etc.) of Kermit-Canfield and Maixner in the past, but because of the sabbatical of one of the founding members and the expertise of the health sciences librarians, it could finally come to fruition.

### Changes for the Biomedical Edition

The health sciences librarians had specific requests for creating a biomedical version of Search&Destroy. They primarily envisioned the game being used with PubMed as the demonstration database but wanted it to be compatible with other common health sciences databases such as Embase and CINAHL. In addition, they also wanted to cover search filters that would appear in a traditional lecture on literature searching from a health sciences librarian.

**Figure 3:**
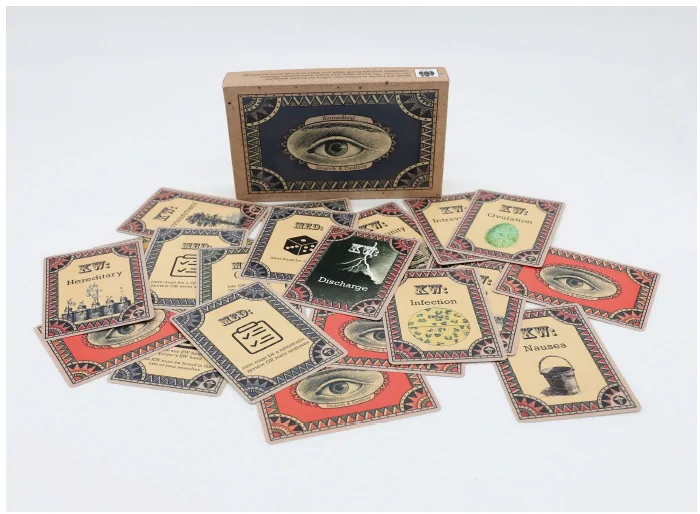
Search&Destroy Biomedical Edition. Photo by Jason Peercy.

In order to accomplish this, the Biomedical Edition required specific adaptations:

1. Additions to and modifications of the keyword deck (search terms) to biomedical/health sciences terms (e.g., skull, suture, pregnancy).
2. Addition of more boolean operator cards to the Play Deck as modification/action cards.
3. Creation of new “Med” Action Cards to the Play Deck with the most commonly used biomedical database filters (e.g., filter for published in the last five years, filter for systematic reviews, filter for RCTs).

All of these changes were meant to make the game more directly applicable to health sciences patrons.

Locating and building the digital materials and original content for Search&Destroy, piecing the parts together, and creating updated packaging and documentation into a material product are a precise, labor intensive, and time-consuming process with a tremendous creative payoff. The physical development of each Search&Destroy edition is extensive, each requiring the sourcing, creation, and assembly of over a hundred cards. Cards are paired with public domain imagery, either icons that direct gameplay in the Play Deck or pictorial images for the Keyword Deck. The pictorial images are majority pre-1900 engravings and etchings with the new additions from period medical books and documents and illustrate each card’s concept (e.g. the “Nausea” Keyword Card is represented by a bucket). Art for a game is important, aesthetics being one of the four main elements of a game [44].

### Playtesting

The game went through three distinct rounds of playtesting:

1. Testing within the group developing the medical version of Search&Destroy.
2. Testing with librarians from Northwell and East Tennessee State University who were not on the development team.
3. Testing with nurse residents.

The first two rounds of testing both led to iterative rounds of improvements. The authors discovered that the game ran too long as we originally designed it and compensated by changing the numbers of different types of action cards – eliminating some that lengthened the game and increasing the number of those that shortened them. The team also changed some of the keyword cards to be more unexpected, since forcing people to choose a non-optimal keyword also shortened the game (and increased the fun).

Nurse residents at Northwell were the first patrons to test the new edition. At Northwell, the librarians teach literature searching to nurse residents often, so they were one of our primary audiences for the game. This playtesting allowed for refining the process of teaching the game in a healthcare setting and doing evaluation with nurses.

**Figure 4:**
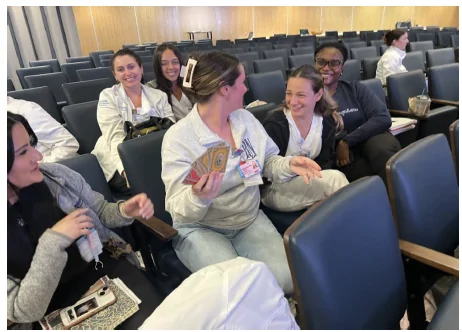
Nurse residents playing Search&Destroy.

### Printing

Search&Destroy is currently produced by the print-on-demand company The Game Crafter, which also acts as a Content Management System for all of the game materials: cards, rulebook, box, etc. [45]. Games are sold at cost and do not turn a profit for the designers. When Search&Destroy is purchased, The Game Crafter prints a copy of the game and ships it. This kind of instant ordering allows game developers to easily make changes to a game. While developing the multiple editions of Search&Destroy this has proven useful to fix mistakes, but it has been better to make major changes in a single large update simply to keep track.

In order to maintain the privacy of the buyer, The Game Crafter provides the game designer only with the city and country name of the location where they are shipping the product. Kermit-Canfield has tracked these locations on Google Maps since Original Search&Destroy was published and has found it satisfying to observe the game’s spread but also frustrating to be unable to perform further assessment with the purchasers of the games.

### Supplemental Teaching Materials

In order to make adopting the game as easy as possible for librarians, the authors also wanted to release a complete package of teaching materials to go along with the game [46]. Ultimately, this came to include a slide deck, a printable handout, a lesson plan, and a best practices sheet for instructors. These were developed and refined in our playtesting with nurse residents.

## DISCUSSION

### Evaluation/Feedback

Because the Northwell librarians frequently teach literature searching to cohorts of nurse residents, we decided to pilot test the Search&Destroy Biomedical Edition with them. The game was overwhelmingly positively received by both the nurse residents and the nurse educators who helped to facilitate the sessions. The students were much more engaged when using the game than previously, when the activity had been a simulated clinical searching exercise. Nurse educator feedback suggested limiting gameplay to under 40 minutes and providing librarians with microphones for improved audio. Nurse resident feedback recommended more thorough gameplay demonstrations and the use of a scoreboard so that teams could more easily track who was ahead.

As the librarians running the pilot, Vitiello and Bohman reflected that the game capitalizes on the fact that health science students are often competitive. Many of the teams enjoyed trash-talking each other, debating the best next move, or occasionally arguing about rules interpretations with the librarians. Even those students who are less engaged with the topic of literature searching don’t want to let their team down in the competition. When teaching with the game, there was often a “light-bulb moment” when one teams search went from a huge number of results (100,000+) to a tiny number (1,000+) over the course of only one turn, which helped teams understand the power of facets and Boolean operators (often to many “oohs” and “aahs”).

Doing a more in-depth assessment of any type of one-shot instruction is challenging. Health professionals’ time is limited, and librarians often only see the students once. It would be very difficult to do an assessment comparing how well game players remembered literature searching lessons longitudinally compared to a control group due to logistical challenges. One potential avenue that the authors did not explore would be a pre- and post-test of literature searching skills for students, primarily because the nurse residency program conducts their own pre- and post-test. However, the authors do not have access to that data.

Based on the positive pilot experience, the game is now integrated into the evidence-based practice component of the nurse residency curriculum. The game is also expanding into other hospitals in the Northwell network through librarian colleagues, and Bohman recently used it successfully to teach Internal Medicine residents as well.

## CONCLUSION/FUTURE DIRECTIONS

Search&Destroy began only as a way to improve the in-class instructional lives of a pair of librarians teaching at a small Midwestern university but has grown to be so much more. As of the time of writing, a Spanish Language Edition (Buscar&Destruír) is nearing completion and the filming of instructional videos for all three editions has begun. An Advanced Boolean Booster Pack will be introduced that will function with any Search&Destroy game, along with subject-specific booster packs that can also be used with the Biomedical Edition. Kermit-Canfield and Maixner have several other higher-ed game projects on deck and are always looking for ways to further the Search&Destroy project. All Search&Destroy games are available for purchase at the Wunderkind Games website.

Despite the value of literature searching skills for medical and health sciences students, librarians often face barriers when working to add these and other information literacy skills to tightly packed curricula. It can be an additional challenge to bring non-traditional learning methods into a library instruction session once established. However, the authors hope that Search&Destroy Biomedical Edition will, in keeping with current trends in health sciences education, be a helpful tool to enliven staid lectures and engage students in active learning through brisk gameplay and friendly competition.

## Data Availability

The game described in this article is available for purchase at cost via The Game Crafter: https://wunderkind-games.com/searchdestroy-medical-edition/.
